# Testing an Evidence-Based Self-Help Program for Infertility-Related Distress: Protocol for a Randomized Controlled Trial

**DOI:** 10.2196/52662

**Published:** 2024-01-18

**Authors:** Jennifer L Gordon, Megan M L Poulter, Ashley A Balsom, Tavis S Campbell

**Affiliations:** 1 Department of Psychology University of Regina Regina, SK Canada; 2 Department of Psychology University of Calgary Calgary, SK Canada

**Keywords:** anxiety, cognitive-behavior therapy, depression, infertility, infertility-related distress, mHealth

## Abstract

**Background:**

Infertility—the inability to achieve pregnancy despite ≥12 months of focused attempts to conceive—is experienced by 1 in 6 couples. Women typically carry a disproportionate share of the psychological burden associated with infertility, experiencing poor quality of life, and 30%-40% experiencing depressive mood or anxiety. Unfortunately, currently available psychological interventions targeting infertility-related distress are associated with modest effects.

**Objective:**

Our team, in collaboration with patient advisors, has designed a self-help intervention for infertility-related distress involving 7 weekly 10-minute videos addressing the cognitive, behavioral, and interpersonal challenges associated with infertility, delivered through a mobile app. A feasibility study suggests that it is well accepted and highly effective in reducing symptoms of anxiety and depressed mood among distressed individuals dealing with infertility. This study represents the next step in this line of research: a fully powered randomized controlled trial comparing the intervention to a waitlist control group.

**Methods:**

We will recruit 170 individuals struggling to become pregnant in Canada or the United States to be randomized to our 7-week self-help program or a treatment-as-usual condition. The primary outcome will be fertility quality of life, while secondary outcomes will include depressive symptoms, anxious symptoms, and relationship quality, assessed before and after the program as well as biweekly for 16 weeks following completion of the program. Self-reported health care use and the presence of diagnosed mood and anxiety disorders, assessed through a structured psychiatric interview, will also be assessed immediately following the intervention and at the 16-week follow-up assessment. Treatment adherence and retention will also be recorded throughout the intervention. Multilevel modeling will compare the intervention arm to the treatment-as-usual condition in terms of all continuous outcomes across the 9 measurement points. Logistic regression will be used to assess the occurrence of mood and anxiety disorders in the 2 treatment arms at the posttreatment assessment as well as at the 16-week follow-up. Sensitivity analyses will examine potential treatment moderators: membership in the LGBTQIA+ (lesbian, gay, bisexual, transgender, queer, intersex, and asexual) communities, baseline fertility quality of life, cultural background, disability status, and pursuit of conception through medical intervention.

**Results:**

We expect our intervention to be more effective than treatment-as-usual in improving all mental health parameters assessed and decreasing health care use related to both mental and reproductive health. Effects are expected to be larger with decreasing baseline quality of life and equally effective regardless of membership in the LGBTQIA+ communities, cultural background, or disability status.

**Conclusions:**

If our intervention is successful, this would suggest that it should be scaled up and made publicly available. The availability of this program would fill an important gap in light of the high rates of psychopathology among those experiencing infertility and considering the current lack of effective psychotherapy approaches for infertility.

**Trial Registration:**

Clinicaltrials.gov NCT06006936; https://classic.clinicaltrials.gov/ct2/show/NCT06006936

**International Registered Report Identifier (IRRID):**

PRR1-10.2196/52662

## Introduction

### Overview

The Canadian Community Health Survey reveals that 16% of Canadian reproductive-aged couples are currently infertile [1], defined as being unable to achieve pregnancy despite ≥12 months of focused attempts to conceive. Although male- and female-factor infertility are equally prevalent, women bear the brunt of the infertility-related burden as treatments require that women monitor their menstrual cycles, attend near-daily ultrasounds, self-inject gonadotropins, and undergo invasive and painful procedures. Women who travel for fertility treatments face additional psychosocial burdens, including schedule disruptions, time off work, and coordination of care among multiple health care providers. It is therefore not surprising that women carry a disproportionate share of the psychological burden associated with infertility, with infertile women consistently reporting lower self-esteem, more instances of depressed mood and anxiety, and lower life satisfaction than their male partners [2]. Lesbian couples pursuing sperm donation experience similar distress, with the intended pregnant individual being at higher risk for depression and anxiety relative to their partner [3].

Around 30%-40% of women presenting for the evaluation of infertility report clinical symptoms of depressed mood and/or anxiety [4-7]. In addition, research from our team [8] suggests that the COVID-19 pandemic has exacerbated distress amid fertility treatment suspensions and delays. Untreated symptoms of depressed mood and anxiety among women with infertility may, in turn, reduce the likelihood of achieving pregnancy, given that psychological burden is the most commonly cited reason for prematurely discontinuing fertility treatments [9]. In a study of 450 couples who were offered 3 government-funded in vitro fertilization (IVF) cycles, 54% did not complete all 3 cycles despite not achieving pregnancy, with “psychological burden” being the primary reason for discontinuing IVF [10]. It is critical that women with infertility who are distressed receive effective mental health treatment to reduce distress and improve conception rates.

Despite high rates of distress among women with infertility, currently available psychological interventions are often ineffective or associated with relatively small benefits. In our recent systematic review of psychological interventions for infertility-related distress [11], we observed that typical interventions are associated with a small beneficial effect on anxiety but a nonsignificant effect on depressive mood, marital quality, or quality of life. Our conclusions confirm findings from a previous meta-analysis [12] and those of a recent review [13] concluding that “a new intervention (targeting infertility-related distress) should be developed.”

### Intervention Development

This trial seeks to test the efficacy of a novel infertility-specific intervention. In developing a treatment that is designed to improve upon the limitations of current approaches, we have used a methodical and evidence-based framework in designing and refining our intervention, using the Medical Research Council [14] and National Institute of Health’s ORBIT Model [15] for trial design interventions as guides. Specifically, we have completed the following milestones to date: (1) completed a systematic review of available interventions, (2) conducted a needs-based assessment using qualitative research methods, (3) carried out an evaluation of potential intervention components, and (4) performed a preliminary test of the acceptability and effect of our newly developed intervention. These steps are described in greater detail below.

#### Systematic Review and Meta-Analysis

As a starting point, we conducted a systematic review and meta-analysis of psychological interventions for infertility [11,16], which included an examination of treatment moderators such as psychotherapeutic approach (eg, mindfulness-based approaches vs cognitive behavioral therapy) and therapy format (eg, self-administered vs group). This process not only confirmed that currently available interventions were largely ineffective but also revealed that neither therapeutic approach nor format significantly impact treatment benefits.

#### Qualitative Needs Assessment

Our team then used semistructured interviews with women with infertility and mental health professionals specializing in infertility to identify the unique aspects of infertility-related distress [17]. Table 1 depicts the themes and subthemes identified. Unique features include the avoidance of infertility reminders (eg, pregnant women and children), excessive cognitive and behavioral focus on one’s infertility to the exclusion of previously enjoyed activities, and negative interactions with loved ones perceived as insensitive.

**Table 1:** Themes and subthemes of infertility-related distress identified through a qualitative research project by our team [17].

Theme 1: anxiety	Theme 2: mood disturbance	Theme 3: threat to self	Theme 4: threat to couple	Theme 5: weakened social support
Anxious rumination	Emotional lability	Unmet expectations for self and one’s future	Differences in coping	Strained romantic relationship
Avoidance of infertility reminders	Helplessness	Shame	Sexual dysfunction	Social stigma
Narrowed focus on infertility-related activities	Emotional exhaustion	Self-blame	Financial stress	Social isolation
Excessive information seeking	N/A^a^	N/A	Disagreement on next steps	Dismissal by health care providers

^a^N/A: not applicable.

In addition to identifying clear psychological targets for our intervention, this study also aimed to clarify which interventions were currently being used by practicing clinicians. Our findings indicated a near-universal use of an eclectic and unstructured approach associated with the widely held opinion that no existing therapeutic approach sufficiently addresses all of the biopsychosocial factors contributing to infertility-related distress.

#### Evaluation of Potential Intervention Components

The next step in our intervention development was to identify and consider all candidate techniques that might effectively target the psychological challenges identified as being common in infertility. To do so, we identified all of the psychotherapeutic approaches endorsed by the American Psychological Association’s Clinical Section as being evidence-based for the treatment of anxiety, mood disorders, relationship difficulties, and chronic illness. The 5 identified approaches were then further broken down into their component procedures, resulting in a total of 14 different psychological techniques. In collaboration with a panel of patient advisors and using lay language, we described how each of these techniques would look when applied to infertility-related distress and what their purpose would be. We then surveyed a total of 644 women from online infertility-specific support groups [18], asking them to rate the perceived usefulness of each of the 14 techniques while asking them to identify up to 5 that were “most liked” and “most hated.” Participants were also given the opportunity to provide written feedback on each of the techniques, such as how they might be better tailored to infertility. We then presented the results of this survey to our panel of patient advisors and collaboratively decided on the content of our intervention. We decided on 6 core modules plus a bonus module, the content of which is described in Textbox 1 [18].

**Chosen modules based on a survey of 644 women with infertility [18] and collaboration with patient advisors. Mean helpfulness ratings for each module, as assessed in a feasibility study of 21 women, are shown.**

**Modules and focus**

- Cognitive restructuring: identifying and challenging the extreme negative thoughts that contribute to a depressive and anxious mood (eg, “In vitro fertilisation will never work”).
- Challenging core beliefs: identifying and challenging unhelpful deep-seated beliefs about themselves, other people, and the world that are perhaps not based on reality (eg, “nothing ever works out for me”). It involves looking for patterns in one’s thinking from the first module.
- Behavioral activation: identifying activities that have been dropped or engaged in less because of an increased focus on infertility. Aim to reintegrate these previously enjoyed activities into their day-to-day lives.
- Sharing your grief: learning about different styles of coping and how clashes in coping styles can lead to conflict within a couple. The individual is instructed on how to engage their partner in a structured conversation about how each can help the other in times of grief, such as following a negative pregnancy test.
- Strengthening your relationship (bonus module): provides evidence-based information about how to better connect with one’s partner in general. Was offered along with Module 4 for those experiencing relationship distress.
- Living your values (ie, stopping avoidance): reflecting on one’s overarching life values and considering how one’s daily actions align with those values. Indirectly addresses avoidance that is common among individuals with infertility (eg, withdrawing from friends and family and avoiding children and pregnant women). Encourages the individual to consider ways in which they can reduce avoidance without worsening their distress.
- Summary or wrap up: providing an overview of the program’s content and encouraging the individual to reflect on what’s been accomplished as well as areas for further development.

When it came time to decide on the format of our intervention, a self-help internet-delivered intervention was chosen for several reasons. First, the findings from our systematic review indicate that self-administered interventions are as effective in improving mental health as other, more costly formats. Second, when asked what format a new intervention should take, 71% (15/21) of women from our needs assessment qualitative study responded that it should be self-delivered through the internet. Third, we reasoned that this format could be most effectively scaled up and made accessible to diverse populations of women and in regions with limited access to psychological services. Thus, in close collaboration with our patient advisors, each module was translated into a 10-minute PowerPoint (Microsoft Corp) slideshow with professional voiceover. A mobile app was then developed to increase accessibility to the modules.

#### Preliminary Testing of Our New Infertility-Specific Intervention

With a new program developed and fully vetted in consultation with our patient advisory panel, we conducted a pilot study of 21 women recruited through local support groups, assessing intervention acceptability [19]. All participants exhibited clinically significant levels of infertility-related distress, as indicated by a Fertility Quality of Life Scale (FertiQoL) score ≤52 [20]. Enrolled through a Zoom (Zoom Video Communications)-administered enrollment session, participants received 1 module per week through email and were asked to view the 10-minute slideshow within 24 hours of receipt. Midweek, they received an email reminder of the homework assignment, encouraging them to apply their homework assignment throughout the week. At the end of each week, participants were asked to rate the extent to which the module and homework were perceived as helpful in lowering their distress (0-10). At baseline and each week, participants completed the Generalized Anxiety Disorder-7 (GAD-7) and 9-item Patient Health Questionnaire (PHQ-9); the FertiQoL was completed at baseline and after the intervention. Each week and in an interview at the end of the study, participants provided written and verbal feedback, respectively, on the intervention.

Of the 21 women enrolled in the study, 2 became pregnant and were removed from the program prematurely (all outcomes assessed until the point of pregnancy were analyzed). Of the remaining 19 women, 15 completed all 6 modules, and 4 completed a portion of the program. Data from all 19 women were included in the analysis. The average helpfulness rating of each module was found to be 7.4 or above out of 10. Fertility quality of life increased by an average of 12 points out of 100, translating to a Cohen *d*=0.9. Large reductions in both mean symptoms of anxiety and depressed mood were observed (pre-to-post Cohen *d*=–1.2 and –1.3, respectively, where effects above 0.8 are considered large), corresponding with clinically meaningful improvements. In addition, 85% of participants experienced a clinically significant decline in either anxious or depressive symptoms (defined as a change of 4 points on the GAD-7 [21] and 5 points on the PHQ-9 [22]).

While the intervention was successful, areas for improvement were identified. For example, homework assignments were modified to include examples of completed homework. Participants reported that our “bonus” relationship module deserved to be a core part of the program, extending it to a total of 7 weeks.

### The Current Trial

Over the last 3 years, our team has carefully designed a self-help intervention for infertility-related distress that is patient-informed and developed using best practices in intervention design. Results from this feasibility study suggest that it is well accepted and effective in increasing quality of life and reducing symptoms of depressed mood and anxiety among women with infertility-related distress. The proposed project, a sufficiently powered randomized controlled trial comparing the intervention to a treatment-as-usual control group, is the next step in this line of inquiry. It is hypothesized that the intervention will result in greater increases in fertility quality of life and relationship quality as well as decreases in symptoms of depressed mood and anxiety relative to a treatment-as-usual condition, and that these improvements will be maintained over a 16-week follow-up assessment period.

If the proposed trial confirms that the intervention is effective in improving quality of life and mental health symptoms among those with infertility, our next step will be to make this program widely available to women, including making the intervention available through YouTube and engaging our collaborating knowledge users and partner organizations to promote it widely. We will also aim to tailor the program for diverse and marginalized underserved groups.

## Methods

### Trial Design

The proposed research is a single-blind randomized controlled trial comparing the above-described self-help program to a waitlist control condition. As the project requires no in-person contact, we will recruit women living throughout Canada and the United States. Fertility quality of life, infertility-related distress, symptoms of depressed mood and anxiety, and relationship quality will be assessed before and after the program, as well as every other week for 16 weeks.

### Treatment Conditions

#### Intervention Condition

Participants will be given access to a 10-minute module video per week through a mobile app created for this trial. Midweek, participants will receive an automated email reminder of the homework assignment for that week, encouraging them to incorporate the homework into their daily lives. Participants will be permitted to engage in any other psychological interventions they wish but will be asked to report other psychological interventions accessed at the end of their participation.

#### Treatment-As-Usual Control Condition

Participants assigned to the control condition will be instructed to continue their pursuits to conceive without accessing the self-help program. They will be permitted to access other psychological resources that are available to them, though, like the intervention condition, they will be asked to report any treatments accessed in the postintervention survey. They will complete the outcome measures at the same time as participants in the treatment condition. Following completion of the study, the control group will be offered the program in the same manner as the treatment group.

In the original funded grant protocol, we had proposed a waitlist control condition in which participants were not permitted to access other mental health services; however, we have since changed the control condition to treatment-as-usual in order to more accurately estimate the real-world effectiveness of the treatment. This change also allows us to open the trial to individuals reporting suicidal ideation because these participants will likely require additional mental health services while participating in the current trial.

### Randomization Scheme

The Clinical Research Support Unit at the University of Saskatchewan will create the randomization scheme and provide the principal investigator with opaque envelopes containing treatment assignments, ensuring that the research team has no control over the assignments. Randomization will take place at the end of each enrollment session, after the baseline surveys have been completed, and will be stratified based on whether a woman is undergoing fertility treatments or attempting to conceive without medical intervention, as this will be a potential moderating variable.

### Protecting Against Sources of Bias

A number of strategies will be used to protect against bias. First, the trial will be registered with clinicaltrials.org before any data collection commences. Second, as described above, the randomization scheme will be created by a third party, and the study research assistants will be instructed to strictly adhere to the randomization protocol without exception. Third, though it is not possible to maintain full blinding of either the participant or research team given the nature of the intervention, all outcomes will be collected by a research assistant who is blind to the participant’s treatment allocation. Fourth, an intent-to-treat approach will be taken in analyzing the results—every effort will be made to continue to collect outcome data on all participants, regardless of whether participants dropped out of the intervention early or not. Final, we will follow the CONSORT-SPI (Consolidated Standards of Reporting Trials for Social and Psychological Intervention Trials) reporting guidelines [23] in reporting the results of the trial, strictly adhering to the original trial protocol. Any deviations will be clearly described and justified.

### Participants

#### Inclusion and Exclusion Criteria

Based on our sample size calculations, we will recruit 170 women, recruited through the web. The inclusion criteria will include the following: is infertile as defined as either actively attempting to conceive for ≥12 months without success or is currently undergoing fertility treatments (eg, ovulation induction medication, IVF, and intrauterine insemination). This definition ensures that this study is inclusive of both individuals who cannot afford fertility treatments and women who are in same-sex couples and cannot conceive naturally. Though the original funded protocol excluded individuals reporting active suicidal ideation, those already receiving psychotherapy, and those with high levels of fertility-related quality of life (FertiQoL above 70), we have since decided to remove these exclusion criteria in order to closely estimate the program’s anticipated real-world effectiveness. Rather than exclude individuals based on baseline quality of life, we will perform secondary analyses, considering baseline quality of life as a treatment moderator.

#### Sex and Gender Considerations

In light of research finding that the intended pregnant individual experiences the most distress in the context of infertility, we will only recruit individuals who have a uterus. However, we will ensure that this study is welcoming to individuals of all gender identities and sexual orientations, as this study will aim to contribute to current knowledge surrounding the psychological experiences of individuals from minority genders, and sexual groups experiencing infertility. This study materials including advertisements, will use inclusive language. Advertisements will not use the word “woman” but instead “individuals attempting to get pregnant but experiencing infertility.” The intervention itself has also been designed with inclusive language, a gender-neutral design, and pictures of individuals from diverse backgrounds and sexual orientations. To ensure adequate diversity among our participants, we will advertise on subreddits specifically targeting members of the LGBTQIA+ (lesbian, gay, bisexual, transgender, queer, intersex, and asexual) community.

#### Participant Screening and Enrollment

Prospective participants will be emailed the link to a web-based eligibility survey. If found to be eligible, they will be asked to provide their contact information, and a research assistant will contact them to schedule an enrollment session through videoconference.

During the Zoom-facilitated remote enrollment session, eligibility will be confirmed, a brief introductory video explaining the study and intervention will be presented, and consent will be obtained. An enrollment session in which visual contact is made will ensure that our recruited participants are not simply “bots” posing as eligible participants. During the session, participants will complete the baseline questionnaires through a link emailed to them by the research assistant. Upon completion of the questionnaires, the research assistant will open an opaque envelope, revealing the participant’s random assignment to either the treatment or control condition. The research assistant will then ask the participant which day of the week they would like to receive their weekly module video (if assigned to the treatment condition) or weekly outcomes survey (if assigned to the control condition).

#### Participant Safety

Participants endorsing suicidal ideation on question 9 of the PHQ-9 at baseline will be permitted to participate in the study but will be informed that their level of risk will be reassessed weekly. Specifically, each week, they will receive a survey question, “Please pick out the one statement that best described how you have been feeling during the past week, including today: (A) I don’t have any thoughts of killing myself. (B) I have thoughts of killing myself, but I would not carry them out. (C) I would like to kill myself, or (D) I would kill myself if I had the chance.” If participants choose either C or D, a message including contact information for 2 suicide hotlines will appear. As well, an automatic notification will be sent to the study therapist, flagging the response. They will then follow up with the participant immediately by phone, at most within 24 hours. If participants endorse A or B, they will simply be allowed to continue with the program.

In addition, the presence of active suicidal ideation (presence of a plan or intent) will be assessed by the researcher during the Zoom-facilitated enrollment session. Those endorsing active suicidal ideation will be referred to additional in-person mental health resources available in their geographic area. They will be given access to the program for their own benefit but will not be enrolled in the study.

### Primary and Secondary Outcomes

#### Overview

Self-reported psychological outcomes will be assessed immediately postintervention (ie, at the end of the 7th week) and every 2 weeks for 16 weeks. Mood and anxiety disorders will be assessed immediately postintervention as well as 16 weeks postintervention. Finally, health care use will be assessed at postintervention week 16. The control group will follow an identical outcome assessment schedule.

#### Demographic and Medical Information

Age, ethnicity, gender identity, sexual orientation, marital and parental status, years of education, income, occupation, reproductive health history, and medications will be assessed using a survey created for this study.

#### Primary Outcome

Fertility-related quality of life was assessed using the 24-item Core FertiQoL [24], yields 4 subscales: mind-body, relational, social, and emotional. High scores on the FertiQoL scale indicate a better quality of life. It is the most widely used infertility-specific measure of quality of life [25] and has been well validated in multiple studies [20]. This primary outcome was chosen in collaboration with our patient advisors as it provides an integrated measure of the emotional, physical, and interpersonal impacts of infertility.

#### Secondary Outcomes

Secondary outcomes will include depressive and anxious symptoms, instances of mood and anxiety disorders, relationship quality, and health care use. Treatment adherence and acceptability will also be assessed.

#### Depressive Symptoms

Self-reported symptoms will be assessed using the PHQ-9 [26], a 9-item measure assessing symptoms in the last 2 weeks that closely parallels the criteria for major depressive disorder as defined by the *Diagnostic and Statistical Manual of Mental Disorders, Fifth Edition* (DSM-5) [27]. Internal consistency coefficient (ICC) has been estimated at α=.89 and test-retest reliability at r=0.84. The PHQ-9 has been shown to be superior to other questionnaires in detecting changes in depressive mood following treatment [28]. Participants endorsing suicidal ideation on item 9 of the PHQ-9 will be required to answer a weekly question.

#### Anxious Symptoms

Self-reported symptoms will be assessed using the GAD-7 [29], a 7-item measure that asks about symptoms in the last 2 weeks and closely parallels the DSM-5 criteria for generalized anxiety disorder. ICC is α=.92. The GAD-7 has been shown to be superior to other questionnaires in detecting change in anxious mood following treatment [30].

#### Mood and Anxiety Disorders

These will be assessed using NetSCID (TeleSage), the computerized version of the Structured Clinical Interview for the DSM-5 (SCID). Though the originally funded protocol proposed to use the Primary Care Evaluation of Mental Disorders (PRIME-MD), we decided to switch to the SCID because of the availability of the computerized version of this interview. The SCID is also the gold standard assessment for psychiatric diagnoses.

#### Relationship Quality

Relationship quality will be measured through the 7-item Relationship Assessment Scale. Internal consistency among individuals with infertility has been found to be high, with α=.83 [31].

#### Health Care Use

The questionnaire on health care consumption and productivity losses for patients with a psychiatric disorder will be administered 16 weeks following the intervention, asking participants to report on health care use in the last 4 months. This survey has shown good agreement with hospital- and employer-confirmed data. An additional section has been added to specifically ask about the receipt of fertility treatments.

#### Treatment Adherence

The total number of minutes spent accessing each moule video will be tracked through the mobile app. Homework completion between the weekly module videos will be tracked using Qualtrics, a web-based survey platform that facilitates scheduled survey distribution, notifications, and reminders. Homework compliance will be further measured at the end of each week through the 12 items contained within the Homework Rating Scale, which assesses comprehension of homework assignments as well as effort spent on assignments [32].

#### Treatment Acceptability

The Credibility Expectancy Questionnaire [33] will be administered at baseline to assess participants’ initial expectations about the intervention. Postintervention, the Treatment Acceptability and Adherence Scale [34] will be used to assess treatment acceptance. The Negative Effects Questionnaire [35] will assess any potential adverse events perceived to be related to the intervention. Final, participants will be invited to provide written feedback about any of the modules or the program as a whole, including suggestions for further improvement and refinement.

#### Outcomes Assessment

A research assistant who is blinded to the participant’s treatment condition will email the participant a link to a web-based survey containing the outcome measures. If a participant fails to complete the survey within 48 hours of receipt, they will receive up to 3 reminders through email, voicemail, and SMS text messaging. Though participants will not be compensated for completing the intervention, they will be compensated US/CAD $10 (or its equivalent depending on their location) for each postintervention survey completed (postintervention +8 biweekly follow-up surveys) and an additional US/CAD $20 for each of the 2 postintervention interviews, for a maximum total of US/CAD $130, to maximize the chances that even those participants who abandoned the intervention prematurely will complete the outcome surveys.

#### Statistical Analyses

Descriptive statistics will examine treatment acceptability outcomes as well as the trial recruitment rate. A 2-tailed *t* test will be used to compare the treatment arms in terms of baseline characteristics, assessing randomization success. A mixed model design using the MIXED procedure in SAS (version 9.4; SAS Institute) applying an intent-to-treat approach will compare the intervention arm to the waitlist control group in terms of FertiQoL, PHQ-9, GAD-7, Copenhagen Multi-Center Psychosocial Infertility Fertility Problem Stress Scale, and Relationship Assessment Scale (RAS) score across the 9 outcome measurement points (ie, at the end of intervention week 7 and biweekly for 16 weeks). Each outcome will be examined in a separate model; subject will be treated as a random effect, and the treatment assignment will be treated as a fixed effect. A repeated statement will identify assessment week as a repeated measure factor. Baseline levels of the outcome will be included as a covariate. This method has been shown to provide optimal statistical power relative to measuring pre- and postintervention outcome change [36]. In using all available data, a mixed model design has also been shown to outperform ad hoc approaches, such as the last-outcome-carried-forward approach [37].

In addition to examining the main effect of treatment assignment on outcomes, the interaction between assignment and assessment week will be examined to determine whether outcomes are maintained across the 9 postintervention measurements. Sensitivity analyses will use a similar approach to examine potential treatment moderators: membership in the LGBTQIA+ communities, baseline FertiQoL score, cultural background, disability status, and pursuit of conception through medical intervention.

The LOGISTIC procedure will assess the occurrence of mood (major or minor depressive episode or persistent depressive disorder) and/or anxiety disorders (generalized anxiety disorder, social anxiety disorder, or panic disorder) in the 2 treatment arms at the posttreatment assessment as well as at the 16-week follow-up.

To limit the family-wise error rate, the Benjamin and Hochberg [38] false discovery rate correction will be applied to all analyses.

#### Power Calculations

Power calculations were performed using G*Power (Axel Buchner) and are focused on the primary outcome, infertility-related quality of life. Based on SDs observed in the population of distressed women with infertility [18], setting α at .05 and power at 80%, a total of 128 participants would be needed to detect a moderate effect size (Cohen *f*=0.25), equivalent to a 6-point difference on the FertiQoL (out of 100) between 2 arms. To allow for a 25% (42/170) dropout rate, we will recruit 170 participants (85 per arm), in line with average completion rate of 82% observed in our meta-analysis of psychological interventions for infertility-related distress and allowing for additional drop-out given considering the 16-week follow-up.

#### Planned Recruitment Rate

We propose to complete the trial within 2 years. The timeline relies on a recruitment rate of 3 participants per week, which we consider to be a highly conservative estimate of what is possible based on our previous experience successfully recruiting participants from this population.

Through our experience in our preliminary work, we have determined that the most successful strategy for recruiting the target population is to advertise through online infertility support or special interest groups—this will therefore be the primary method used to recruit for this study. We have found these groups to be very willing to share our research, and their members are extremely receptive as well as highly likely to be eligible to participate. We have also had great success in recruiting individuals from LGBTQIA+ communities attempting to conceive through IVF. In an ongoing study specifically targeting this population, we approached a pair of social media influencers (a lesbian couple who regularly share their experiences of undergoing IVF) who enthusiastically shared our project with their followers. Within 2 days, we had received over 400 entries in our eligibility survey.

In addition to providing large pools of highly engaged, eligible participants, one important advantage of web-based recruitment is that samples tend to be much more diverse in terms of race, education, and income relative to studies that recruit through fertility clinics, the patients for which are disproportionately high-income. While web-based recruitment can increase the risk of recruiting noneligible individuals posing as eligible participants, the use of a face-to-face Zoom enrollment session greatly reduces this risk. Our research team is also experienced in identifying suspicious survey responses.

### Ethical Considerations

This study has been reviewed and approved by the University of Regina Ethics Board (REB #2023-210) as well as registered on ClinicalTrials.gov (NCT06006936). All prospective participants will provide informed written consent before enrolling in the trial.

To protect participant confidentiality, all participant data, including both interview data and questionnaire data, will be saved under ID numbers only, with no identifying information attached. Only the research team will have access to the collected survey data. The team will maintain a document associating participant names with their anonymous subject numbers. This document will be password-protected, opened only on encrypted devices, and stored separately from the rest of the data.

Participants will receive US $10 for each postintervention and follow-up survey completed and US $20 for each of the 2 postintervention interviews, for a maximum total of US $130.

## Results

Recruitment will begin in January 2024 and continue for approximately 1.5 years. All data are expected to be collected by January 2026. Results will be uploaded on the ClinicalTrials.gov website shortly thereafter.

## Discussion

### Significance of the Study

It is expected that participants assigned to the Coping with Infertility program will exhibit improved fertility quality of life as well as depressive and anxious symptoms, with moderate to large effect sizes. We also expect rates of clinical mood and anxiety disorders as well as self-reported health care use to be lower among participants randomized to the treatment arm. Baseline fertility quality of life is furthermore expected to moderate the effect of treatment such that effect sizes will increase with decreasing baseline fertility quality of life. Based on the pilot study results, we expect adherence and retention to be favorable. If our hypotheses are confirmed, these findings would suggest that the Coping with Infertility program is more effective than currently available psychological interventions for infertility. Indeed, a recent meta-analysis by our team identified 58 randomized controlled trials testing psychological interventions for infertility and found that, with the exception of trials conducted in the Middle East, interventions were associated with only small psychological benefits, highlighting the need for more effective interventions [16]. The self-help nature of the Coping with Infertility program also likely makes it more cost-effective than individual psychotherapy, which typically costs US $100-$200 per session.

If our intervention proves effective, we will aim to make our mobile app publicly available through the Apple Store and Google Play Store. Decreased health care use in the treatment arm relative to the treatment-as-usual arm would provide a strong rationale for seeking government funding to upkeep the Coping with Infertility mobile app, which would allow us to make the program available free of charge. We would provide flyers and posters to fertility clinics across North America, to be posted in clinic waiting rooms and physician offices. We will reach out to relevant professional societies and nonprofit organizations, asking them to include the app as a mental health resource listed on their website. Online forums relevant to infertility will also be contacted and asked to share information related to the app. A YouTube channel will be created to house all of the weekly module videos along with a professionally produced animated explainer video introducing the intervention and describing the results from the trial supporting its efficacy. Final, we will publish our findings in open-access journal articles in respected scientific journals.

In addition to disseminating the Coping with Infertility program as a stand-alone intervention, it may also be worthwhile to pair it with other traditional mental health resources. For example, future research pairing the Coping with Infertility program with infertility support groups, or with individual psychotherapy may help target a broader audience of individuals experiencing infertility-related distress who wish to benefit from peer or therapist support. Translating the content of the program into a workbook format may also appeal to a subset of the target population.

### Limitations

First, access to the Coping with Infertility program is contingent upon internet access; research participants may therefore not include individuals who do not have such access, such as those who cannot afford internet access or those living in remote communities. Second, due to the nature of the intervention, it is impossible to conduct this trial as a double-blind, randomized trial. Third, health care use will be self-reported and therefore may not capture use as accurately as hospital and clinic records.

### Conclusions

This study will test a self-help program for infertility-related distress through a mobile app. If the intervention proves effective, it will provide a highly cost-effective and accessible mental health resource for those struggling with the mental health impacts of infertility. This will fill an important gap in light of high rates of psychopathology among those experiencing infertility and considering the current lack of effective psychotherapy approaches for infertility.

## Appendix

Multimedia Appendix 1 Peer review report from the Canadian Institutes of Health Research.

## Abbreviations

CONSORT-SPI: Consolidated Standards of Reporting Trials for Social and Psychological Intervention Trials
DSM-5: Diagnostic and Statistical Manual of Mental Disorders, Fifth Edition
FertiQoL: Fertility Quality of Life Scale
GAD-7: Generalized Anxiety Disorder-7
ICC: internal consistency coefficient
IVF: in vitro fertilization
LGBTQIA+: lesbian, gay, bisexual, transgender, queer, intersex, and asexual
PHQ-9: 9-item Patient Health Questionnaire
PRIME-MD: Primary Care Evaluation of Mental Disorders
SCID: Structured Clinical Interview for the DSM-5
