# Supplementary material for: Testing an Evidence-Based Self-Help Program for Infertility-Related Distress: Protocol for a Randomized Controlled Trial
Source: JMIR Res Protoc. 2024 Jan 18;13:e52662. doi: 10.2196/52662 (PMC10835586; doi:10.2196/52662)
Supplement: Multimedia Appendix 1 [file resprot_v13i1e52662_app1.pdf]

Canadian Institutes of Health Research/Instituts de recherche en santé du Canada

Notice of Recommendation/Avis de recommandation

Application Number/Numéro de la demande: 486085
Committee Code/Code du comité: PB1

Applicants/Candidats: Dr. Jennifer Lee Gordon

With/Avec: Dr. T. Campbell   Ms. M. Schultz   Ms. L. Stultz

Institution paid/ Établissement payé: University of Regina (Saskatchewan)

Title/Titre: Testing an Evidence-Based Self-Help Program for Infertility-Related Distress

Primary Inst./Inst. principal: Neurosciences, Mental Health and Addiction
Other Related Inst./ Autres inst. connexes:

Competition /Concours: Project Grant
September/Septembre 14, 2022

Number in competition/Nbre de demandes dans le concours: 1899

**Peer Review Committee Recommendation, for your information and use/
Recommandation du comité d'examen par les pairs, pour fins d'information et d'utilisation:**

**Committee/Comité:**	Psychosocial, Sociocultural & Behavioural Determinants of Health
**Number reviewed/ Demandes examinées:**	44
**Application rank within the committee/ Rang de la demande dans le comité:**	3
**Percent Rank within the committee / Rang en pourcentage au sein du comité:**	95.35%
**Rated / Cote:**	4.46
**Recommended Term/ Durée recommandée:**	3 years/ans 0 months/mois
**Recommended average annual operating amount/ Montant annuel moyen recommandé pour le fonctionnement:**	$116,667
**Recommended equipment amount/ Montant recommandé pour les appareils:**	$0

This document is for information only.
An application rated below 3.50 is ineligible for CIHR funding. For applications rated 3.50 and above, please note that it is the application's rank within the peer review committee that determines whether it is funded, rather than its absolute rating. The final funding decision will be communicated in the Notice of Decision.

Document à titre d'information seulement.
Une demande cotée en dessous de 3,5 n'est pas admissible au financement des IRSC. En ce qui a trait aux demandes cotées 3,50 ou plus, veuillez noter que l'on détermine l'attribution des fonds en fonction du classement obtenu au sein du comité d'examen par les pairs plutôt qu'en fonction du classement absolu. La décision finale relative au financement sera communiquée dans l'Avis de décision.

**Review Type / Type d'évaluation:**	Reviewer 1 / Évaluateur 1
**Name of Applicant / Nom du chercheur:**	Gordon, Jennifer
**Application No. / Numéro de demande:**	486085
**Agency / Agence:**	CIHR/IRSC
**Competition / Concours:**	Project Grant/Subvention Projet
**Committee / Comité:**	Psychosocial, Sociocultural & Behavioural Determinants of Health/Déterminants psychosociaux, socioculturels et comportementaux de la santé
**Title / Titre:**	Testing an Evidence-Based Self-Help Program for Infertility-Related Distress

Adjudication Criteria/Critères de sélection

Initial Score/Cote Initiale: 4.4

Top/Bottom Selection/Groupe supérieur/inférieur

- (●) **Top/Groupe supérieur**
- ( ) **Bottom/Groupe inférieur**

**Review Type / Type d'évaluation:**	Reviewer 1 / Évaluateur 1
**Name of Applicant / Nom du chercheur:**	Gordon, Jennifer
**Application No. / Numéro de demande:**	486085
**Agency / Agence:**	CIHR/IRSC
**Competition / Concours:**	Project Grant/Subvention Projet
**Committee / Comité:**	Psychosocial, Sociocultural & Behavioural Determinants of Health/Déterminants psychosociaux, socioculturels et comportementaux de la santé
**Title / Titre:**	Testing an Evidence-Based Self-Help Program for Infertility-Related Distress

Summary of Application/Résumé de la demande:

One in six couples experience infertility, which places a disproportionate physical and psychological burden on the intended pregnant partner. A substantial proportion of women experiencing infertility experience clinically significant depression or anxiety; however, access to specialized mental health resources is limited in Canada. Moreover, a recent knowledge synthesis suggested that existing interventions are ineffective for infertility-related distress. This research team has therefore developed of a cost-effective self-help intervention for infertility-related distress, that is patient informed and founded on scientific evidence. A feasibility study provided initial support for the acceptability and effectiveness of this intervention. The proposed work is a randomized trial evaluating the efficacy of this novel self-help intervention compared to wait-list control in a sample of 170 individuals with infertility with a 16-week follow up.

**Review Type / Type d'évaluation:**	Reviewer 1 / Évaluateur 1
**Name of Applicant / Nom du chercheur:**	Gordon, Jennifer
**Application No. / Numéro de demande:**	486085
**Agency / Agence:**	CIHR/IRSC
**Competition / Concours:**	Project Grant/Subvention Projet
**Committee / Comité:**	Psychosocial, Sociocultural & Behavioural Determinants of Health/Déterminants psychosociaux, socioculturels et comportementaux de la santé
**Title / Titre:**	Testing an Evidence-Based Self-Help Program for Infertility-Related Distress

Strengths and Weaknesses/Forces et faiblesses:

Strengths

This resubmission boasts numerous strengths:

-The focus of the current investigation is strongly supported by the prevalence and impact of infertility-related distress and the lack of available interventions

-The development of this novel self-help intervention was based on well-established frameworks and included engagement with women with infertility and other key knowledge users

-The pilot investigation of feasibility and acceptability meaningfully informed the self-help intervention content

-The involvement in knowledge users including those with lived or living expertise throughout this initiative to date, including in the current proposal, is a clear strength

-The design is rigorous, with well-validated measures, well-specified analytic approach, and close attention to bias (e.g., randomization processes, allocation concealment, intent-to-treat analyses, etc.)

-The knowledge translation plan is multi-faceted and detailed.

-Numerous letters of support endorse the feasibility of the proposed research and associated knowledge mobilization.

-The proposal were responsive to many comments, e.g., increased attention to gender and sexual diversity throughout the introduction, longer follow-up duration, clarified analytic approach, healthcare utilization and costs assessment, French language content, etc.

-The NPI has a strong track record of funding and publication in this field and in patient-oriented research, and the research team has a successful history of collaboration and includes a range of trainees, researchers across career stages, knowledge users, and lived expertise.

Weaknesses

-Some key comments were attended to to a more limited degree – e.g., hypotheses were specified only briefly or imprecisely

-Greater attention to other forms of diversity would be beneficial, as well as social determinants of health. Indeed, intersectionality not touched on, despite the impact of features such as cultural background, disability status, etc. on service access, engagement, and outcomes.

-It would be useful to incorporate safety considerations given the nationwide recruitment of participants.

**Review Type / Type d'évaluation:**	Reviewer 1 / Évaluateur 1
**Name of Applicant / Nom du chercheur:**	Gordon, Jennifer
**Application No. / Numéro de demande:**	486085
**Agency / Agence:**	CIHR/IRSC
**Competition / Concours:**	Project Grant/Subvention Projet
**Committee / Comité:**	Psychosocial, Sociocultural & Behavioural Determinants of Health/Déterminants psychosociaux, socioculturels et comportementaux de la santé
**Title / Titre:**	Testing an Evidence-Based Self-Help Program for Infertility-Related Distress

Budget Recommendation/Recommandation budgétaire:

The proposed budget appears reasonable.

Please indicate your appraisal of the integration of sex as a biological variable as a strength, weakness, or not applicable to the proposal./Prière de sélectionner une option pour donner votre évaluation de l'intégration du sexe comme variable biologique en tant que point fort ou point faible de la proposition, ou en tant qu'élément non applicable à la proposition.

- ○ **Strength/Point fort**
- ○ **Weakness/Point faible**
- ◉ **Not applicable/Non applicable**

Please indicate your appraisal of the integration of gender as a socio-cultural determinant of health as a strength, weakness, or not applicable to the proposal./Prière de sélectionner une option pour donner votre évaluation de l'intégration du genre comme déterminant socioculturel de la santé en tant que point fort ou point faible de la proposition, ou en tant qu'élément non applicable à la proposition.

- ◉ **Strength/Point fort**
- ○ **Weakness/Point faible**
- ○ **Not applicable/Non applicable**

**Review Type / Type d'évaluation:**	Reviewer 1 / Évaluateur 1
**Name of Applicant / Nom du chercheur:**	Gordon, Jennifer
**Application No. / Numéro de demande:**	486085
**Agency / Agence:**	CIHR/IRSC
**Competition / Concours:**	Project Grant/Subvention Projet
**Committee / Comité:**	Psychosocial, Sociocultural & Behavioural Determinants of Health/Déterminants psychosociaux, socioculturels et comportementaux de la santé
**Title / Titre:**	Testing an Evidence-Based Self-Help Program for Infertility-Related Distress

Sex and/or Gender Considerations/Notions de sexe et/ou de genre:

Sex considerations are not relevant to the current proposal due to the focus on adults with a uterus. The proposal is inclusive of gender identities however as well as sexual orientation.

**Review Type / Type d'évaluation:**	Reviewer 2 / Évaluateur 2
**Name of Applicant / Nom du chercheur:**	Gordon, Jennifer
**Application No. / Numéro de demande:**	486085
**Agency / Agence:**	CIHR/IRSC
**Competition / Concours:**	Project Grant/Subvention Projet
**Committee / Comité:**	Psychosocial, Sociocultural & Behavioural Determinants of Health/Déterminants psychosociaux, socioculturels et comportementaux de la santé
**Title / Titre:**	Testing an Evidence-Based Self-Help Program for Infertility-Related Distress

Adjudication Criteria/Critères de sélection

Initial Score/Cote Initiale: 4.2

Top/Bottom Selection/Groupe supérieur/inférieur

- (●) **Top/Groupe supérieur**
- ( ) **Bottom/Groupe inférieur**

**Review Type / Type d'évaluation:**	Reviewer 2 / Évaluateur 2
**Name of Applicant / Nom du chercheur:**	Gordon, Jennifer
**Application No. / Numéro de demande:**	486085
**Agency / Agence:**	CIHR/IRSC
**Competition / Concours:**	Project Grant/Subvention Projet
**Committee / Comité:**	Psychosocial, Sociocultural & Behavioural Determinants of Health/Déterminants psychosociaux, socioculturels et comportementaux de la santé
**Title / Titre:**	Testing an Evidence-Based Self-Help Program for Infertility-Related Distress

Summary of Application/Résumé de la demande:

The investigators propose to conduct a one-arm randomized trial comparing a 7-week internet-delivered self-help program to waitlist control for N = 170 women with infertility related distress. Participants will include those who have significant infertility related distress and have not conceived in 12 months + or are receiving infertility treatment. Participants with elevated suicidality or who are concurrently receiving psychological treatment will be excluded. Outcome measures will include depression, anxiety, and quality of life.

**Review Type / Type d'évaluation:**	Reviewer 2 / Évaluateur 2
**Name of Applicant / Nom du chercheur:**	Gordon, Jennifer
**Application No. / Numéro de demande:**	486085
**Agency / Agence:**	CIHR/IRSC
**Competition / Concours:**	Project Grant/Subvention Projet
**Committee / Comité:**	Psychosocial, Sociocultural & Behavioural Determinants of Health/Déterminants psychosociaux, socioculturels et comportementaux de la santé
**Title / Titre:**	Testing an Evidence-Based Self-Help Program for Infertility-Related Distress

Strengths and Weaknesses/Forces et faiblesses:

Strengths

The team is a strength, including a productive NPA, who is an associate professor and Canada Research Chair in biopsychosocial determinants of women's health, has published extensively (and in high impact journals; current h-index = 17) on this topic and who has taken the lead on the development of the self-help intervention. The Co-PIs are well-positioned to provide relevant expertise in both the conduct of RCTs for medical populations and in counselling. The project also includes patient advisors, which will enhance acceptability and clinical relevance.

The proposal is well written, organized, and clear. The applicants provided an excellent snapshot of the development of this self-help intervention to date. Findings based on development work, which was done systematically according to ORBIT and MRC standards, show strong effects, acceptability and feasibility. They also have justified design decisions with strong rationales and discussed barriers and how to get around them.

The applicants made a strong case for the potential impact of this work, with infertility having a disproportionate impact on women (of differing sexual orientations), and extant research on treatments for infertility-related distress showing effects that do not appear clinically significant. Also, community clinicians appear to use eclectic and non evidence-based approaches. The use of an e-delivered, self-guided approach will facilitate dissemination and availability.

The outcome measures are well-validated and relevant.

Statistical analyses are appropriate.

The KT plan was strong.

The applicants made a strong case for feasibility of recruitment.

Overall, this is a very well-conceived project with the potential for strong and unique health impact.

Weaknesses

This can't be addressed now, but in the preliminary work, the main question for participants had to do with which modules were most effective at reducing their distress. That's one important goal, but the ability to tolerate distress or improve quality of life could be others.

Type-O on p.4 where the applicants suggest the intervention will result in "greater decreases in fertility quality of life…."

I appreciated the concern about including individuals beyond a certain level of suicidality, given the waitlist condition and need for treatment other than self-guided self-help. That said, I'd like to see more creative ways to include people who are suicidal. I believe some research has suggested that placebo controls are not necessarily detrimental or unethical with suicidal populations. I wonder if future research might examine ways to include suicidal people, such as by including ancillary crisis management or treatment. The intervention is only 7 weeks, so it seems like this could be manageable.

Although measures were generally on target and appropriate, I might have wanted to see some structured diagnostic assessment. I also think it could be valuable to include measures of relationship satisfaction or adjustment, as the relationship context could have a strong impact on psychological adjustment to infertility and could become part of the treatment.

**Review Type / Type d'évaluation:**	Reviewer 2 / Évaluateur 2
**Name of Applicant / Nom du chercheur:**	Gordon, Jennifer
**Application No. / Numéro de demande:**	486085
**Agency / Agence:**	CIHR/IRSC
**Competition / Concours:**	Project Grant/Subvention Projet
**Committee / Comité:**	Psychosocial, Sociocultural & Behavioural Determinants of Health/Déterminants psychosociaux, socioculturels et comportementaux de la santé
**Title / Titre:**	Testing an Evidence-Based Self-Help Program for Infertility-Related Distress

Budget Recommendation/Recommandation budgétaire:

I’d recommend keeping the budget as is.

Please indicate your appraisal of the integration of sex as a biological variable as a strength, weakness, or not applicable to the proposal./Prière de sélectionner une option pour donner votre évaluation de l'intégration du sexe comme variable biologique en tant que point fort ou point faible de la proposition, ou en tant qu'élément non applicable à la proposition.

- ○ **Strength/Point fort**
- ○ **Weakness/Point faible**
- ◉ **Not applicable/Non applicable**

Please indicate your appraisal of the integration of gender as a socio-cultural determinant of health as a strength, weakness, or not applicable to the proposal./Prière de sélectionner une option pour donner votre évaluation de l'intégration du genre comme déterminant socioculturel de la santé en tant que point fort ou point faible de la proposition, ou en tant qu'élément non applicable à la proposition.

- ◉ **Strength/Point fort**
- ○ **Weakness/Point faible**
- ○ **Not applicable/Non applicable**

Sex and/or Gender Considerations/Notions de sexe et/ou de genre:

Sex is not relevant as all participants are female, but gender is relevant, and the investigators have described attempts to recruit a sample that's diverse in terms of sexual orientation. I might have wanted to see projections of how many people with different sexual orientations are expected. Although it's not the focus of the project, it could be worth examining subgroups, and future development of this treatment could entail more specific adaptations or refinements for those populations if necessary.

**Review Type / Type d'évaluation:**	Reviewer 3 / Évaluateur 3
**Name of Applicant / Nom du chercheur:**	Gordon, Jennifer
**Application No. / Numéro de demande:**	486085
**Agency / Agence:**	CIHR/IRSC
**Competition / Concours:**	Project Grant/Subvention Projet
**Committee / Comité:**	Psychosocial, Sociocultural & Behavioural Determinants of Health/Déterminants psychosociaux, socioculturels et comportementaux de la santé
**Title / Titre:**	Testing an Evidence-Based Self-Help Program for Infertility-Related Distress

Adjudication Criteria/Critères de sélection

Initial Score/Cote Initiale: 4.5

Top/Bottom Selection/Groupe supérieur/inférieur

- (●) **Top/Groupe supérieur**
- ( ) **Bottom/Groupe inférieur**

**Review Type / Type d'évaluation:**	Reviewer 3 / Évaluateur 3
**Name of Applicant / Nom du chercheur:**	Gordon, Jennifer
**Application No. / Numéro de demande:**	486085
**Agency / Agence:**	CIHR/IRSC
**Competition / Concours:**	Project Grant/Subvention Projet
**Committee / Comité:**	Psychosocial, Sociocultural & Behavioural Determinants of Health/Déterminants psychosociaux, socioculturels et comportementaux de la santé
**Title / Titre:**	Testing an Evidence-Based Self-Help Program for Infertility-Related Distress

Summary of Application/Résumé de la demande:

•One in six couples experience infertility, with a disproportionate mental health burden on the pregnant partner. This team has recently developed a feasible, well accepted, highly feasible evidence-based, patient-informed, cost-effective 7-week self-help intervention for infertility-related distress. They propose a fully powered single-blind waitlist RCT with 170 individuals to assess effectiveness of the intervention on fertility QoL as well as depressive symptoms, anxious symptoms, infertility-related distress, and relationship quality. Follow-up continues to 16-weeks post-intervention (nine measurement points in total). If successful, applicants will share the intervention on YouTube and promote broadly.

**Review Type / Type d'évaluation:**	Reviewer 3 / Évaluateur 3
**Name of Applicant / Nom du chercheur:**	Gordon, Jennifer
**Application No. / Numéro de demande:**	486085
**Agency / Agence:**	CIHR/IRSC
**Competition / Concours:**	Project Grant/Subvention Projet
**Committee / Comité:**	Psychosocial, Sociocultural & Behavioural Determinants of Health/Déterminants psychosociaux, socioculturels et comportementaux de la santé
**Title / Titre:**	Testing an Evidence-Based Self-Help Program for Infertility-Related Distress

Strengths and Weaknesses/Forces et faiblesses:

•This is a relatively small team, with a Co-A Clinical Psychologist and two Knowledge Users. NPA is an ECR CRC who began work in this domain with a SHRF Patient-Oriented Leader Award ($250K), and past work includes foundation work identifying infertility-related coping strategies and distress as well as a scoping review and meta-analyses of existing interventions (n=9 publications).

•The team provides a compelling case for the proposed research, having conducted many different foundation studies using a POR approach. It would be good to understand the generalizability of extant literature and the team’s research, to help better inform a balanced approach to potential expansion to other geographies and sociodemographics.

•Easily scalable intervention, with effective feasibility study complete. Final modifications to the intervention are being informed by the patient of panel advisors.

•Some revisions to the study design were not carried through all sections (e.g. Section 2.1 still states only one month of post-program weekly follow-ups versus the biweekly for 16 weeks in other sections; Section 2.14 still states “Considering the short follow-up time of one month”).

•Good randomization process outlined.

•How is stratification by fertility treatment versus no medical intervention operationalized in terms of sample size? This is important given plans for a sensitivity analysis based on this factor.

•Discussion of bias in the study does not address possible selection bias concerns, and no description of participant recruitment strategies is provided.

•Is there no eligibility criteria related to age and English-language? Is there a requirement to be in a relationship (e.g. thinking about participants who may be engaging in fertility treatments as a sole parent), especially given that module has been made “core” and that relationship quality is a secondary outcome.

•Good plan for Zoom-facility face-to-face screening and enrolment. Does the study team have a sense of recruitment attrition across these steps/phases?

•Good rationale for primary outcome and secondary outcomes.

•How long are the surveys and is the honoraria appropriate?

•The applicants justify and provide evidence to the treatment adherence based on their feasibility study and meta-analysis, but not much to justify a further estimated 8% drop-out during the 16-week of follow-up now planned.

•Analysis plan could more clearly restate commitment to use intent-to-treat approach.

•Equipoise for waitlist control, especially given the extended follow-up time (recognizing the extended FU time came from previous reviewers)? The plan for patient safety/monitoring could be expanded beyond just adverse events. Is a single final data analysis justified?; more rationale should be provided.

•Sub-groups analyses outlined in Section 2.18 have measures that are not fully described or congruent with measures in Section 2.8.

•Strong response to reviewers, and revisions made accordingly.

•What is the KT plan if the trial is not successful?

•In the project information section, applicants indicate that their application does not involve Indigenous peoples nor address TCPS2 Chapter 9, which is not further described in the proposal.

•In the future, NPA may want to consider additional budget items such as time for a provider to support those reporting suicidality (as could be significant time investment for PI), additional staff time for attrition during recruitment, costs related to more representative or diversity-focused sampling strategies, benefits costs for staff, additional staff costs to support KT phase of the project, computers and software for PI and PhD student, sufficient conference travel budget (is $2500 sufficient for the PI to cover registration, travel, accommodations, etc.), etc.

•Good to see matching funding provided by USask for teaching release for PI as this increases feasibility.

**Review Type / Type d'évaluation:**	Reviewer 3 / Évaluateur 3
**Name of Applicant / Nom du chercheur:**	Gordon, Jennifer
**Application No. / Numéro de demande:**	486085
**Agency / Agence:**	CIHR/IRSC
**Competition / Concours:**	Project Grant/Subvention Projet
**Committee / Comité:**	Psychosocial, Sociocultural & Behavioural Determinants of Health/Déterminants psychosociaux, socioculturels et comportementaux de la santé
**Title / Titre:**	Testing an Evidence-Based Self-Help Program for Infertility-Related Distress

Budget Recommendation/Recommandation budgétaire:

No budget comments

Please indicate your appraisal of the integration of sex as a biological variable as a strength, weakness, or not applicable to the proposal./Prière de sélectionner une option pour donner votre évaluation de l'intégration du sexe comme variable biologique en tant que point fort ou point faible de la proposition, ou en tant qu'élément non applicable à la proposition.

- (●) **Strength/Point fort**
- ( ) **Weakness/Point faible**
- ( ) **Not applicable/Non applicable**

Please indicate your appraisal of the integration of gender as a socio-cultural determinant of health as a strength, weakness, or not applicable to the proposal./Prière de sélectionner une option pour donner votre évaluation de l'intégration du genre comme déterminant socioculturel de la santé en tant que point fort ou point faible de la proposition, ou en tant qu'élément non applicable à la proposition.

- (●) **Strength/Point fort**
- ( ) **Weakness/Point faible**
- ( ) **Not applicable/Non applicable**

**Review Type / Type d'évaluation:**	Reviewer 3 / Évaluateur 3
**Name of Applicant / Nom du chercheur:**	Gordon, Jennifer
**Application No. / Numéro de demande:**	486085
**Agency / Agence:**	CIHR/IRSC
**Competition / Concours:**	Project Grant/Subvention Projet
**Committee / Comité:**	Psychosocial, Sociocultural & Behavioural Determinants of Health/Déterminants psychosociaux, socioculturels et comportementaux de la santé
**Title / Titre:**	Testing an Evidence-Based Self-Help Program for Infertility-Related Distress

Sex and/or Gender Considerations/Notions de sexe et/ou de genre:

•These are sufficient, focusing on relevant biological factor (uterus) for eligibility despite generally referring to women in the rest of the proposal, an inclusive recruitment design, and plan for subgroups analyses. However, the applicants do not indicate if they are sufficiently powered to do this. Further, what about other intersectional forms of diversity? Might the applicants consider a specific SBGA-informed research aim/question?

Review Type/Type d'évaluation: SO Notes /Notes de l'agent scientifique
Name of Applicant/Nom du chercheur: Gordon, Jennifer Lee
Application No./Numéro de demande: 486085
Agency/Agence: CIHR/IRSC
Competition/Concours: 2022-09-14 Project Grant/Subvention Projet
Committee/Comité: Psychosocial, Sociocultural & Behavioural Determinants of Health/Déterminants psychosociaux, socioculturels et comportementaux de la santé
Title/Titre: Testing an Evidence-Based Self-Help Program for Infertility-Related Distress

---

Assessment/Évaluation:

Strengths (including SGBA considerations):

The committee was enthusiastic about this proposal and the applicant's revisions based on past feedback.

The team has demonstrated expertise and patient engagement with end knowledge users, including pilot work.

Well-written proposal with clearly outlined rationale and objectives.

Knowledge translation plan was seen as a strength.

SGBA a strength of this proposal, although there was discussion on whether the proposal was adequately powered to address all analyses and whether inclusivity and diversity could be further explored. Cultural intersectionality was also discussed by the committee.

Potential impact was seen as high, as was scalability potential in a critical area that needs to be addressed.

Recruitment plan was seen as feasible – to a potential downside! May need to consider plan for what to do if overwhelmed by strong recruitment strategies and likelihood of high public interest to participate in this study.

Measures sound.

Weaknesses (including SGBA considerations):

All weaknesses considered minor.

Hypotheses could have been more specific.

Considerations encouraged for finding ways to include those who are suicidal.

Review Type/Type d'évaluation: SO Notes /Notes de l'agent scientifique
Name of Applicant/Nom du chercheur: Gordon, Jennifer Lee
Application No./Numéro de demande: 486085
Agency/Agence: CIHR/IRSC
Competition/Concours: 2022-09-14 Project Grant/Subvention Projet
Committee/Comité: Psychosocial, Sociocultural & Behavioural Determinants of Health/Déterminants psychosociaux, socioculturels et comportementaux de la santé
Title/Titre: Testing an Evidence-Based Self-Help Program for Infertility-Related Distress

Assessment/Évaluation:

Extension of follow-up period length could have been more justified.

Eligibility criteria should clearly state it is English-speaking language only.

Budget:

No concerns.

****

Note: The final rating of the application, provided in the Notice of Recommendation (NOR) and Notice of Decision (NOD), is the averaged rating of the peer review committee members following the discussion of the application during the committee meeting, and therefore may differ from the ratings provided by the assigned reviewers in their respective reviews.

Remarque : La cote définitive de la demande, qui apparaît dans l'avis de recommandation et l'avis de décision, représente la moyenne des cotes accordées par les membres du comité d'évaluation par les pairs après avoir débattu de la demande à la réunion du comité. Elle peut donc différer de celle donnée par les évaluateurs dans leur évaluation respective.

````````````````````````````````````````````````````````````````````````````````````````````````````````````````````````
````````````````````````````````````````````````````````````````````````````````````````````````````````````````````````
